# Characterization of Cabernet Sauvignon Wines by Untargeted HS-SPME GC-QTOF-MS

**DOI:** 10.3390/molecules27051726

**Published:** 2022-03-07

**Authors:** Alejandra Chávez-Márquez, Alfonso A. Gardea, Humberto González-Rios, Luz Vazquez-Moreno

**Affiliations:** Centro de Investigación en Alimentación y Desarrollo A.C., Carretera Gustavo Enrique Astiazarán Rosas, No. 46, Col. La Victoria, CP., Hermosillo 83304, Sonora, Mexico; alejandra.chavez@estudiantes.ciad.mx (A.C.-M.); gardea@ciad.mx (A.A.G.); hugory@ciad.mx (H.G.-R.)

**Keywords:** untargeted method validation, wine, Mexican wine, metabolomics, HS-SPME GC-QTOFMS

## Abstract

Untargeted metabolomics approaches are emerging as powerful tools for the quality evaluation and authenticity of food and beverages and have been applied to wine science. However, most fail to report the method validation, quality assurance and/or quality control applied, as well as the assessment through the metabolomics-methodology pipeline. Knowledge of Mexican viticulture, enology and wine science remains scarce, thus untargeted metabolomics approaches arise as a suitable tool. The aim of this study is to validate an untargeted HS-SPME-GC-qTOF/MS method, with attention to data processing to characterize Cabernet Sauvignon wines from two vineyards and two vintages. Validation parameters for targeted methods are applied in conjunction with the development of a recursive analysis of data. The combination of some parameters for targeted studies (repeatability and reproducibility < 20% RSD; linearity > 0.99; retention-time reproducibility < 0.5% RSD; match-identification factor < 2.0% RSD) with recursive analysis of data (101 entities detected) warrants that both chromatographic and spectrometry-processing data were under control and provided high-quality results, which in turn differentiate wine samples according to site and vintage. It also shows potential biomarkers that can be identified. This is a step forward in the pursuit of Mexican wine characterization that could be used as an authentication tool.

## 1. Introduction

As demand increases, knowledge about food and beverage quality and authenticity also grows. Untargeted metabolomics approaches are emerging as powerful tools [1,2,3]. Metabolomics comprise the analysis of all metabolites (low-molecular-weight molecules) present in a cell, organism or system, accomplished preferentially, in a single analysis [4]. Experimentally, metabolomics analysis represents a great challenge because of its premise, particularly untargeted methods with the purpose of measuring as many metabolites as possible, while chemical identity is not necessary before data acquisition [5]. Targeted method guidelines are constantly updated; however, metabolomics method validation is complicated and revised guidelines of minimum reporting standards for untargeted studies are needed [6,7]. Consequently, the metabolomics community is encouraging the implementation and communication of quality assurance and quality control in untargeted metabolomics studies [8,9,10,11,12].

In recent years, metabolomics approaches have been applied in wine science for quality determination in order to evaluate the influence of different enological practices, microbial fermentation behavior and terroir. However, most have not reported the method validation, quality assurance and/or quality control applied, as well as assessment through the metabolomics-methodology pipeline [13,14,15,16,17,18,19,20,21], for more examples see [2]. The untargeted metabolomics-methods pipeline consists of four main steps, as proposed by Brown et al. [22]: (1) experimental design and metadata capture; (2) data preprocessing; (3) cleaned data; and (4) data to knowledge, or as recently expressed: (1) sample collection and processing; (2) data acquisition; (3) data processing; and (4) data interpretation [5,23]. Consequently, in order to obtain significant and reproducible data, each step needs to be controlled. Several guidelines have been reported to ensure method validation in different matrices [5,10,24,25,26]; recently, a pipeline for an untargeted HS-SPME GC-qTOF/MS-method workflow to analyze wine samples was proposed [27]. Here, we describe a recursive analysis as an alternative method for data mining.

While Mexico is considered the oldest wine-growing region in the Americas, knowledge of its viticulture, enology and wine science remains scarce [28]. Currently, Baja California State produces 75% of Mexican wine [29], mainly in Ensenada, where the second-oldest Mexican vineyard was planted in Santo Tomás Valley. Wine consumption and production in Mexico has increased in recent years, although Mexican wine remains insufficient to meet demand [30]. Hence, in 2018 an initiative to increase wine production and foster research in wine sciences was approved [31], as well as a commercial brand (Vino Mexicano) to promote Mexican wine quality. Hopefully, the characterization of wines will provide the industry with a tool to regulate and guarantee quality and authenticity of Mexican wines [32]. Thus, untargeted metabolomics approaches emerge as a suitable tool to support this initiative. Therefore, the objective of this study is to characterize and differentiate Cabernet Sauvignon wines from two different vintages and vineyards using an untargeted HS-SPME GC-QTOF-MS-validated method.

## 2. Results

### 2.1. Method Validation and Data Acquisition

The untargeted analysis objective is to determine as many metabolites as possible; therefore, highly repeatable and reproducible data are required. However, it has been reported that it is not clear how exhaustive and reliable current raw data processing is [12]. Therefore, although complicated, it is clear that method validation and quality analysis are needed. In order to validate the method, guidelines for targeted methods were included (Repeatability, Reproducibility, Linearity, LOD and LOQ). Although these parameters had to be determined for each targeted metabolite, in untargeted methods it is suggested to select metabolites that are present in samples, have similar chemical properties and molecular mass and are distributed along the runtime of the acquisition method [10]. Based on this, the chemical standards α-Pinene, β-Pinene, p-Cymene and 2-Undecanone were selected (Supplementary Materials Table S1).

**Figure 1:**
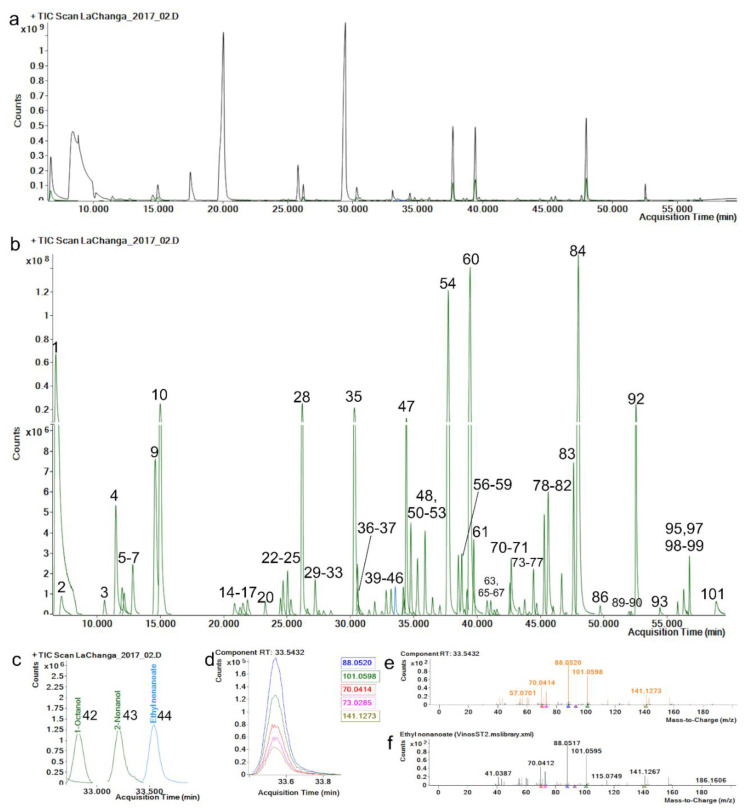
(**a**) Total ion chromatogram (TIC, black) and (**b**) extracted components (green) identified (see Table 1) by manual recursive analysis. (**c**) Enlarged ethyl nonanoate peak. (**d**) Ethyl nonanoate ion peaks; each color represents an ion, exact mass is represented in the same color. (**e**) Ethyl nonanoate mass-spectrum fragmentation-pattern comparison, orange: acquired spectrum, (**f**) black: library spectrum.

The repeatability and reproducibility of the extracted component area of each level and metabolite were <20.0% RSD (Supplementary Materials Table S1), complying with recommended criteria [33]. The retention-time (RT) reproducibility of all standards was <0.5% RSD, where maximum variation (SD) was of 0.09 min (5.4 s); this minimal variation enhances alignment across samples and identification by default. Hence, a match-factor penalty is applied in method identification if the RT variation is greater than 12 s. Match-factor reproducibility was <2.0% RSD, which can be related to mass-fragmentation spectrum stability which depends on mass-spectrum comparison and mass accuracy. Mass accuracy in the five most abundant fragments of each standard was <5 ppm (Figure 1e,f), which allowed match factors greater than 80 on all metabolite identifications.

Selectivity was assessed with the method capability to successfully discriminate between isomers α-Pinene and β-Pinene (136.125 g/moL) by retention time and mass fragmentation spectrum. Selectivity is an important quality to enable component extraction in the data-processing phase [10]. LOD concentrations were below 0.2 ng/L for each standard and LOQ were 2.5 ng/L (Supplementary Materials Table S1), indicating a high sensitivity in the method for detecting low-abundance components. Even though these parameters cannot be used to quantify other metabolites in untargeted methods [34], this method still provides an overview of metabolites’ chromatographic behavior. Interestingly, p-Cymene and 2-Undecanone could be used as an internal control for SPME fiber’s life span. As a sign of fiber deterioration, p-Cymene splits in two chromatographic peaks and 2-Undecanone abundances greatly decrease (data not shown).

An advantage of using a wine-spiked pool as matrix for method validation was that 74 compounds were identified, allowing the calculation of their extracted-area reproducibility ≤ 15.0% RSD (Supplementary Materials Table S2). Figure 1a shows a typical total ion chromatogram (TIC) of wine components; however, components present in most abundant chromatographic peaks could not be identified because of ion saturation and/or ion peak aberrancy. Thus, a method with split desorption must be performed to identify these metabolites. Since our interest resides in the low-abundance (Figure 1b and Table 1) metabolites present in wines, and the method was able to separate and extract them (Figure 1c,d), we decided to work with a splitless method to analyze samples. Consequently, method validation demonstrated that the extracted components’ area, RT and match factor were reproducible and unaffected by the concentration required for successful data processing/mining, data identification and data interpretation/analysis. 

### 2.2. Quality Control

**Figure 2:**
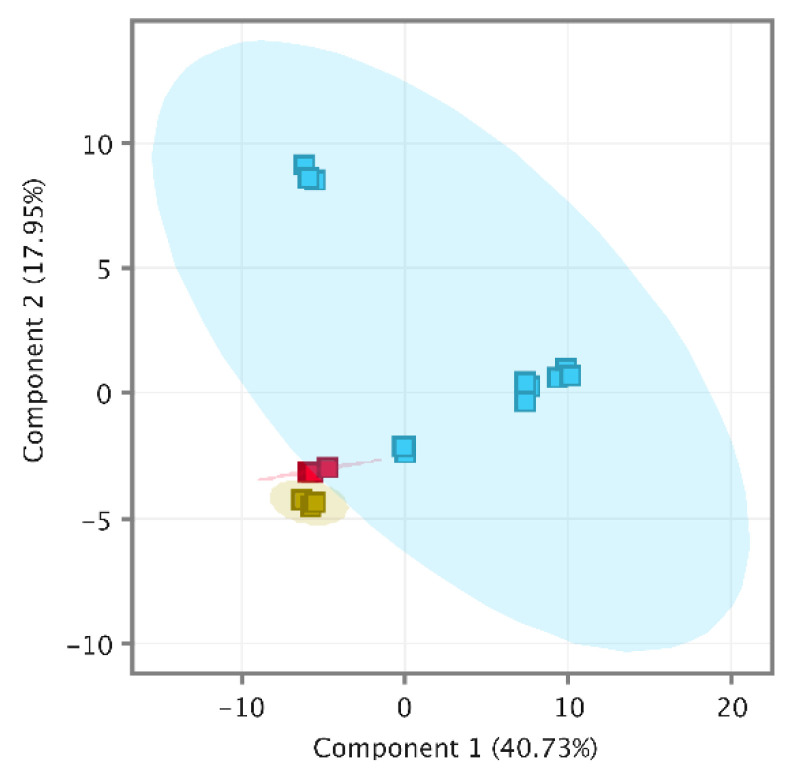
PCA of quality-control samples, PW (yellow), PWS (red) and samples (blue).

Recursive analysis successfully identified 74 compounds in a pooled wine (PW) and 76 in spiked pooled wine (PWS). Interestingly, isobutyl acetate (116.1583 g/moL) was identified in the PW (RT 10.65 min) but not in spiked samples. It seems that the method could not extract the isobutyl acetate component peak from the spiked α-pinene component peak. Moreover, it appears to include a p-cymene carryover of 0.06 ng/L which is less than 20% of LOQ, the acceptance criteria recommended by the FDA [33] for targeted analysis. Overall quality-control analysis was performed using MPP (MassHunter Workstation, Agilent Technologies, Santa Clara, CA, USA) by importing CEF files of PW, PWS and wine samples. The quality-control PCA (Principal Component Analysis) included a total of 109 entities and was clustered tightly out of all QC samples from wine samples, as shown in Figure 2; therefore, the data set was considered to be of high quality [5] and we proceeded to data interpretation.

### 2.3. Wine Characterization

**Figure 3:**
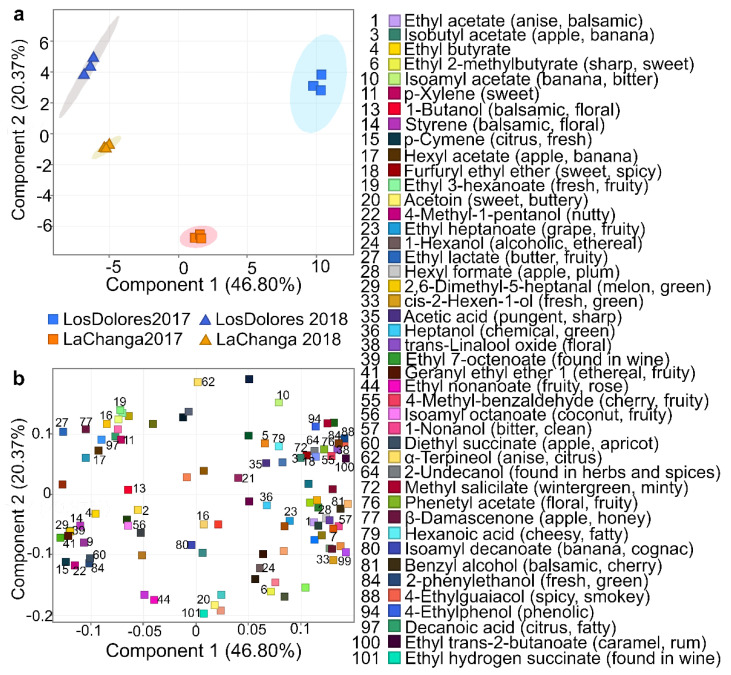
Principal component analysis (PCA). (**a**) grouping vineyards (blue: Los Dolores, yellow: La Changa) and vintages (■: 2017, ▲: 2018); and (**b**) compound distribution, identifying those that contributed greatly to sample differentiation.

Recursive analysis extracted and identified 77, 75, 78 and 73 metabolites in wines from La Changa 2017 and 2018 and Los Dolores 2017 and 2018, respectively (Table 1). PCA included 101 metabolites (Table 1), where the first three components explained 86.71% of total variance (data not shown); furthermore, using the first two components (67.02% of total variance) allowed the clustering of wines by vineyard and vintage (Figure 3a) with a metabolite distribution shown in Figure 3b (PCA loadings). To elucidate PC1 and PC2’s meaning, a closer glance at metabolites near to wine-clustering areas was required; PC1 appears to be related to variables depending upon vintage, while PC2 allows the separation of wines by typology, and therefore is associated with wine quality or/and sensorial profile.

Regarding PC2 (Figure 3), some of its positive loadings such as 4-ethylguaiacol (compound #88 Table 1) and 4-ethylphenol (#94) contribute with undesirable aromas and have been reported in wines affected with Brettanomyces [35]. However, 2-undecanol (#64), 4-methylbenzaldehyde (#55), furfuryl ethyl ether (#18), methyl salicylate (#72) and trans-linalool oxide (#38) have been associated with spicy notes or found in spices, with roasted nuts, cooked beef and blackberry aromas; isoamyl acetate (#10) has banana and balsamic notes and α-terpineol (#62) has anise and citrus. At the same time, some of its negative PC loadings—metabolites such as monoethyl succinate (#101), ethyl nonanoate (#44), acetoin (#20), diphenyl ether (#87) and isobutyl hexanoate (#26)—have desirable sensorial properties and are reported as sweet and fruity [36].

Further analysis on PCA-loading distribution (Figure 3b) showed that metabolites at PC1- and PC2-negative loadings have fruity and citrus descriptors; those at PC1-negative and PC2-positive loadings are described as fresh, sweet, floral and fruity; while those at PC1-positive and PC2-negative loadings are predominantly floral and sweet notes. PC1- and PC2-positive loadings are less desirable, with descriptors such as alcoholic, balsamic and phenolic [36]. Based on these descriptors it could be inferred that the 2018 vintages from both vineyards have fruitier, more citrus, sweeter and fresher notes than the 2017 vintage, and Los Dolores 2017 presents floral notes. According to these results, La Changa 2017 could be the most-balanced wine as it is positioned almost at the center of the PCA (Figure 3a); however, sensorial analysis is required to confirm these assumptions. In addition, some putatively identified (level 2) and unknown (level 4) components have potential use as biomarkers for vineyard and vintage classification; consequently, their elucidation is required [7].

**Figure 4:**
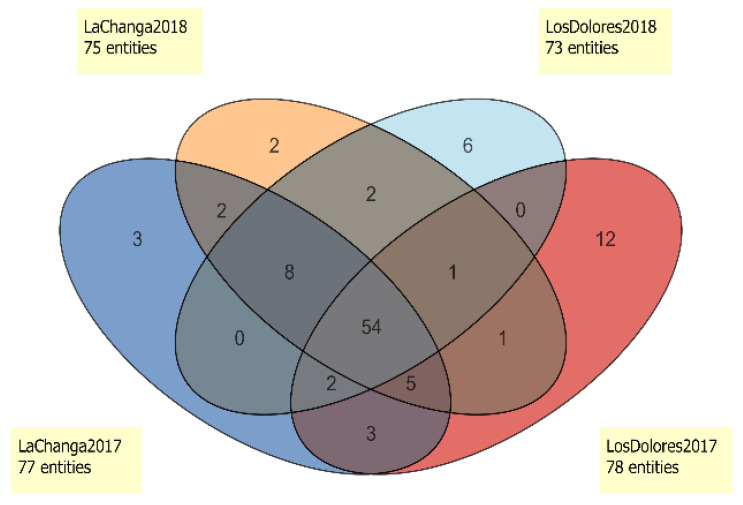
Venn diagram of entities present in La Changa 2017 (dark blue), La Changa 2018 (orange), Los Dolores 2017 (red) and Los Dolores 2018 (light blue).

Additional data analysis (Figure 4) showed five potential markers for vintage differentiation. Compounds cis-2-Hexen-1-ol (#33), Unknown 38.9824 (RI 1432; ions 43.0543, 71.0852, 57.0699, 70.0774, 55.0543 *m*/*z*; C6H12O) and Unknown 41.3476 (RI 1754; ions 163.1114, 145.1008, 164.1161, 73.0645, 45.0335 *m*/*z*; C11H12O3) were unique to 2017 wines. Ethyl lactate (#27) and octyl ether (#68) were only present in 2018 wines (Figure 4 and Table 1). Interestingly, 54 compounds are shared by vineyards and vintages, which ideally, could indicate a metabolomic fingerprint of Santo Tomás Valley; however, extensive sampling and further analysis is needed to conclude this. Nevertheless, 24 of those compounds (Table 2) were decisive in vintage differentiation (*p* < 0.05). Styrene (#14), methyl octanoate (#30), β-damascenone (#77), decanoic acid (#97) and Unknown 51.9854 (RI 2072, ions 85.0290, 69.0696, 41.0386, 71.0488, 43.0179, C15H26O3) were distinctive in the 2018 vintage; meanwhile, 19 compounds were distinctive for 2017. Additionally, in the 2018 vintage, ethyl 3-methylbutyrate (#7), 1-octanol (#42), ethyl phenylacetate (#73) and 2,4-di-tert-buthylphenol (#99) decreased 3- to 5-fold (FC > 3.0, *p* < 0.001).

Interestingly, 2,4-di-tert-buthylphenol (#99) was 5-fold higher in the 2017 vintage than in 2018. To our knowledge, it has not been reported in Cabernet Sauvignon wines; however, was detected with the same abundance in red and white wines from Portugal [37]. Furthermore, Marselan wines (Cabernet Sauvignon × Grenache varieties) inoculated with *S. cerevisiae* presented higher concentrations of 2,4-di-tert-buthylphenol than in spontaneously fermented wines [38]. Persimmon-inoculated wines showed similar behavior [39]. This compound has antifungal and antioxidant characteristics [40] but no aromatic properties have been reported yet. Moreover, the compound was first detected at the end of alcoholic fermentation in the 2017 vintage and increased after malolactic fermentation (data not shown). Although produced by non-Saccharomyces yeasts [41] and lactic-acid bacteria [40], 2,4-di-tert-buthylphenol could be a potential marker in vintage differentiation as microbial terroir cannot be discarded.

The Venn diagram (Figure 4) showed that 54 compounds were present in all wines and enabled the selection of unique compounds for each one. Los Dolores 2017 wine presented 12 distinctive compounds (4-methyl benzaldehyde, trans-linalool oxide (furanoid), ethyl trans-2-butanoate, furfuryl ethyl ether, 4-ethylguaiacol, 4-ethylphenol, methyl salicylate, 2-undecanol and Unknowns 13.2265; 29.3449; 48.8990 and 52.3099), and Los Dolores 2018 wines presented only six (p-xylene, diphenyl ether, isobutyl hexanoate, ethyl ether hexanoic acid and Unknown 16.5912). La Changa 2017 wines presented three unique compounds (ethyl hydrogen succinate, acetoin and Unknown 54.4274), and the 2018 vintage presented two (1-butanol and 4,1,1-dimethyl-trans-cyclohexanol). However, compounds such as ethyl nonanoate and Unknown 54.4273 (RI 2194, ions 149.0441; 105.0692; 104.0615; 133.0128: 150.0449 *m*/*z*, formula C8H7NO2) were found only in wines from La Changa vineyard. This set of compounds could be used as potential markers to identify wines from La Changa vineyard, although as stated before, a larger sample size must be analyzed for confirmation.

Reports have estimated that 62% of metabolites present in wine remain unidentified and target metabolomics cannot resolve this drawback [2], thus generating a free library with reliable data of unknown metabolites (accurate mass spectrum, RI, RT and potential formula) that could enable their rapid identification. This feature should be added as part of the minimum reporting standard procedure [7] to enhance probability and move identification levels upward. Furthermore, it will provide a robust and comprehensive workflow report that could improve reproducibility of results and the exchange of experimental data among research groups [5].

## 3. Conclusions

Metabolomics studies urgently require establishing guidelines for validation of untargeted methods, particularly for complex matrices such as beverages. Here, we used parameters for targeted experiments combined with recursive analysis of data for quality assurance to show that both chromatographic and spectrometry-processing data were under control and complied with certain guidelines. During validation, an accurate mass library, VinoST2.mslibrary.xml, was created, and included the retention index, retention time and CAS number of metabolites that were putatively identified, and the exact mass and molecular formula of those classified as unknowns.

Recursive analysis of metabolite data and PCA successfully differentiated Cabernet Sauvignon wines from two vineyards and two vintages and gave an approximation of their aromatic notes. In addition, potential markers of vineyard and vintage were pointed out, and a profile of 54 compounds was described in all Cabernet Sauvignon wines from Santo Tomás Valley. This effort constitutes an advance in the pursuit of Mexican wine characterization that could be used as an authentication tool.

## 4. Materials and Methods

### 4.1. Samples

Cabernet Sauvignon wines of vintages 2017 and 2018 from two different vineyards were collected from 55,000 L stainless steel tanks, bottled (750 mL, sealed with natural cork) and stored horizontally at room temperature until sample processing. Vineyard-management practices of La Changa and Los Dolores vineyards, from Bodegas de Santo Tomás (Ensenada, B.C., Mexico, 31°34′ N, 116°24′ W, elevation 180 m.a.s.l.) were the same in both sites and along those two vintages, as well their vinification process. For quality-control (QC) purposes a subset of samples was pooled (vintages 2015, 2016 and 2017; PW), aliquoted and stored at −50 °C until needed.

### 4.2. Data Acquisition

Samples were analyzed using a 7890B GC System (Agilent Technologies, Santa Clara, CA, USA) coupled to a 7200 mass spectrometer with quadrupole-time-of-flight (MS-qTOF) (Agilent Technologies, Santa Clara, CA, USA), with an autosampler PAL3 System (CTC Analytics AG, Zwingen, Switzerland) and a head-space solid-phase micro-extraction (HS-SPME) module with 50/30 μm DVB/CAR/PDMS Stable Flex Supelco fiber (Agilent Technologies, Santa Clara, CA, USA) [42]. Three grams of NaCl were added to 10 mL of sample in a 20 mL amber vial sealed with an aluminum cap and an 18 mm blue PTFE/silicone septum (Agilent Technologies, Santa Clara, CA, USA), as described [43]. Modified parameters for extraction [44], separation [45] and detection are summarized in Table 3. Mass calibration was performed at the beginning and after running three samples, to ensure mass accuracy.

### 4.3. Method Validation

To validate the data-acquisition method, repeatability, reproducibility, linearity and limits of detection (LOD) and quantification (LOQ), the chromatographic standards α-Pinene and p-Cymene from Honeywell Fluka™ (Morristown, NJ, USA) and, β-Pinene (99%) and 2-Undecanone (99%) from Sigma-Aldrich (St. Louis, MO, USA) were used. Validation parameters were performed in a pooled-wine (PW) matrix to prevent matrix interferences. Concentration range of α-Pinene and β-Pinene was 1.56 (L1) to 25.00 (L5) ng/L with 1:1 factor, while p-Cymene and 2-Undecanone was 0.31 (L1) to 5.00 ng/L (L5) with the same factor. Repeatability was determined using a five-level curve by triplicate on day one. Reproducibility was calculated with a three-level curve (L1, L3 and L5), also in triplicate, on the second day of work. Mean, standard deviation (SD) and relative standard deviation (%RSD) were calculated for each level to determine repeatability and reproducibility. Linearity was determined by the correlation coefficient (*r*) of five-level standard curves and PW as a blank sample (matrix sample without standards). LOD and LOQ were determined from ten injections of L1 in three different days (Supplementary Materials, Table S1) and calculated using Agilent MassHunter WorkStation Quantitative Analysis version 10.0 (Agilent Technologies, Santa Clara, CA, USA).

### 4.4. Quality Control

Quality control was assessed by monitoring pooled samples of Cabernet Sauvignon wines from Santo Tomás. Every batch sequence of injections included a PW, PWS with standards at L4 concentration (Supplementary Materials Table S1) and samples from both vintages and vineyards. Injections were randomized, analyzed in triplicate (Supplementary Materials Table S3) and processed by recursive analysis as described in the Data Processing section.

### 4.5. Data Processing/Mining and Identification

#### 4.5.1. Data Processing/Mining

Data processing/mining of raw data was an exhaustive and crucial step for untargeted analysis; this process must generate a holistic and reliable representation of the metabolites present in each sample [5]. Data processing was performed in two steps in order to generate a recursive analysis (as pretreatment to ease data interpretation/analysis of complex matrices) using Agilent MassHunter WorkStation Unknowns Analysis software version 10.0. All data acquired were converted to the SureMass format (only data acquired in profile mode can be converted). First step for recursive analysis was to extract and identify most of components in the QC pool to create an internal library (see Internal library); second step was recursive analysis (described later). Component extraction was performed using SureMass deconvolution with a retention-time (RT) window factor of 300, a 5 SNR (signal-to-noise ratio), extraction window of ±10 ppm, threshold of 25% in component shape, a minimum of four ion peaks for extraction and a maximum of 10 ion peaks to store. Area and height filters were not applied because the aim of this study was to also include minor compounds, which resulted in an exhaustive manual/visual analysis of ion-peak shapes. Extracted components were identified with Accurate Mass Flavors Database [46] and NIST 17, as described below.

#### 4.5.2. Retention Index

Retention indices (RI) were calculated using 50 ng/L C8-C40 Alkanes calibration standard (Sigma-Aldrich, St. Louis, MO, USA). Liquid injection (1 μL) in manual mode was used to improve the signal acquired. Acquired data was processed as indicated above and identified with NIST 17 library, then exported in library format. Agilent MassHunter WorkStation Library Editor 10.0 was used to activate only “Compound name”, “CAS#”, “Retention Index” and “Retention Time” columns, in that order, and saved in a CSV (comma-separated values) format to create the RT calibration file, which contains alkanes RI to be used in recursive analysis to calculate the RI of unknown components.

#### 4.5.3. Internal Library

For data reduction and to decrease false positives and false negatives, QC-pooled sample was analyzed to generate an internal library for recursive analysis. Components extracted were identified with Accurate Mass Flavors Database [46] and NIST 17. Identification method (level 2, as proposed by Sumner et al. [33] included spectral search with a minimum match factor of 70, performing an exact-mass comparison, starting at 30 *m*/*z*, with accuracy < 20 Δppm. Once automatic identification was carried out, manual/visual analysis was completed (components identified as fiber and column materials were eliminated). Putatively identified compounds were assigned when ion peaks, mass-fragmentation spectrum and RI matched (ΔRI < 30) libraries’ components. If one of these parameters did not comply, the component was exported as library file and identified as Unknown + RT (min). With this method, an exact-mass library, VinoST2.mslibrary.xml (available at https://www.ciad.mx/VinosMxDB, accessed: 6 November 2019), was created and includes RT and RI of a total of 93 compounds, with 25 of them identified as Unknowns (*m*/*z* shown in Supplementary Materials, Table S4).

#### 4.5.4. Recursive Analysis

VinoST2.mslibrary.xml library was added to recursive-analysis method using RT as a match factor with a trapezoidal penalty range of 18 s and a penalty-free range of 12 s, in order to align components across samples. Libraries Flavors-14-mslibrary.xml and NIST 17 were added and used without RT as a match factor to identify components not present in QC sample, using the same parameters applied to internal library creation and then adding them to it. RT calibration file was also included to calculate RIs. In order to identify a given compound with the internal library, the component of interest had to be present in the three replicates and match the compound fragmentation spectra, RI and RT (Figure 1). Once all samples were analyzed and their compounds identified, AllBestHits script was run to export the data in CEF format (Compound Exchange Format).

### 4.6. Data Interpretation/Analysis

Data interpretation/analysis was performed using Agilent MassHunter WorkStation Mass Profiler Professional (MPP) version 15.0. Identified components’ data were imported and grouped by vineyard (La Changa and Los Dolores) and vintage (2017 and 2018), considering a 2 × 2 factorial design, then transformed with the median of the baseline of all samples and used to perform a principal-component analysis (PCA) on all entities and samples where variance and covariance matrix method was used. A Venn diagram was performed with entities’ lists of wines from both vineyards and vintages. From this, all entities in both vineyards were selected to perform a moderated *t*-test (*p*-value cut-off of 0.05 and Benjamini–Hochberg as multiple testing correction, FC > 1.1) comparing vintages. Moreover, a 2-way ANOVA was performed, pairing conditions between vintages and vineyards.

## Figures and Tables

**Table 1:** Compounds present in both vintages’ and vineyards’ red wines.

#	RT	RI	Compound	Id	La Changa				Los Dolores						CAS Number	Formula
					2017 Ab (RelAb%)	2018 Ab (RelAb%)	FC		2017 Ab (RelAb%)	FC		2018Ab (RelAb%)	FC			
1	6.7	900	Ethyl Acetate **	2	9.2 × 10^8^ (68)	7.6 × 10^8^ (67)	↓	1.2	1.2 × 10^9^ (52)	↑	1.3	5.4 × 10^8^ (27)	↓	1.7	141-78-6	C4H8O2
2	7.2	918	Methyl Alcohol	2	1.7 × 10^7^ (1)	1.5 × 10^7^ (1)	↓	1.2	1.4 × 10^7^ (0.6)	↓	1.2	1.7 × 10^7^ (1)	↓	1.0	67-56-1	CH4O
3	10.6	1028	Isobutyl acetate *	2	6.4 × 10^6^ (0.5)	6.4 × 10^6^ (0.6)	↓	1.0	8.2 × 10^6^ (0.4)	↑	1.3	6.4 × 10^6^ (0.5)	↑	1.0	110-19-0	C6H12O2
4	11.5	1051	Ethyl butyrate ***	2	6.5 × 10^7^ (5)	5.6 × 10^7^ (5)	↓	1.2	4.6 × 10^7^ (2)	↓	1.4	6.9 × 10^7^ (5)	↑	1.1	105-54-4	C6H12O2
5	12.0	1064	1-Propanol	2	2.3 × 10^7^ (2)	1.4 × 10^7^ (1)	↓	1.6	2.7 × 10^7^ (1)	↑	1.2	2.7 × 10^7^ (2)	↑	1.2	71-23-8	C3H8O
6	12.2	1068	Ethyl 2-methylbutyrate	2	1.3 × 10^7^ (1)	2.9 × 10^6^ (0.3)	↓	4.5	1.2 × 10^7^ (0.5)	↓	1.1	ND	-	-	7452-79-1	C7H14O2
7	12.8	1084	Ethyl 3-methylbutyrate *	2	2.5 × 10^7^ (2)	5.3 × 10^6^ (0.5)	↓	4.8	2.2 × 10^7^ (1)	↓	1.1	5.7 × 10^6^ (0.4)	↓	4.4	108-64-5	C7H14O2
8	13.2	1093	Unknown 13.2265	2	ND	ND	-	-	1.7 × 10^6^ (0.1)	-	-	ND	-	-	-	C9H20O2
9	14.6	1127	Isobutyl alcohol	2	1.1 × 10^8^ (8)	1.2 × 10^8^ (10)	↑	1.1	9.5 × 10^6^ (4)	↓	1.2	1.1 × 10^8^ (9)	↑	1.0	78-83-1	C4H10O
10	15.0	1135	Isoamyl acetate	2	2.4 × 10^8^ (18)	2.3 × 10^8^ (20)	↓	1.0	2.8 × 10^6^ (12)	↑	1.2	2.7 × 10^8^ (18)	↑	1.1	123-92-2	C7H14O2
11	15.3	1143	p-Xylene	3	ND	ND	-	-	ND	-	-	7.5 × 10^5^ (0.1)	-	-	106-42-3	C8H10
2	16.6	1170	Unknown 16.5912	4	ND	ND	-	-	ND	-	-	1.1 × 10^6^ (0.1)	-	-	-	C10H16
13	17.3	1182	1-Butanol	2	ND	4.6 × 10^6^ (0.4)	-	-	ND	-	-	ND	-	-	71-36-3	C4H10O
14	20.8	1261	Styrene **	2	5.4 × 10^6^ (0.4)	5.4 × 10^6^ (0.5)	↑	1.0	2.9 × 10^6^ (0.1)	↓	1.9	7.2 × 10^6^ (0.6)	↑	1.3	100-42-5	C8H8
15	21.2	1268	p-Cymene	1	3.4 × 10^6^ (0.3)	7.6 × 10^6^ (0.1)	↓	4.4	ND	-	-	1.3 × 10^6^ (0.1)	↓	2.6	99-87-6	C10H14
16	21.4	1273	Isoamyl butyrate	2	7.1 × 10^6^ (0.5)	3.6 × 10^6^ (0.3)	↓	2.0	4.6 × 10^6^ (0.2)	↓	1.5	5.5 × 10^6^ (0.4)	↓	1.3	106-27-4	C9H18O2
17	21.8	1284	Hexyl acetate **	2	7.5 × 10^6^ (0.6)	8.6 × 10^6^ (0.8)	↑	1.1	7.6 × 10^6^ (0.3)	↑	1.0	1.0 × 10^7^ (0.8)	↑	1.3	142-92-7	C8H16O2
18	22.7	1301	Furfuryl ethyl ether	2	ND	ND	-	-	1.6 × 10^6^ (0.1)	-	-	ND	-	-	1000450-02-5	C7H10O2
19	22.8	1306	Ethyl 3-hexenoate	3	ND	ND	-	-	ND	-	-	2.7 × 10^6^ (0.2)	-	-	2396-83-0	C8H14O2
20	23.3	1319	Acetoin	2	4.7 × 10^6^ (0.4)	ND	-	-	ND	-	-	ND	-	-	513-86-0	C4H8O2
21	23.9	1328	Unknown 23.8754	2	ND	2.3 × 10^6^ (0.2)	-	-	2.3 × 10^6^ (0.1)	-	-	ND	-	-	-	C10H18O
22	24.5	1343	4-Methyl-1-pentanol	2	6.1 × 10^6^ (0.5)	3.3 × 10^6^ (0.3)	↓	1.9	ND	-	-	3.3 × 10^6^ (0.3)	↓	1.9	626-89-1	C6H14O
23	24.6	1345	Ethyl heptanoate	2	9.6 × 10^6^ (0.7)	6.5 × 10^6^ (0.6)	↓	1.5	8.5 × 106 (0.4)	↓	1.1	6.7 × 10^6^ (0.5)	↓	1.4	106-30-9	C9H18O2
24	25.1	1356	1-Hexanol	2	1.5 × 10^7^ (1)	6.5 × 10^6^ (0.6)	↓	2.3	1.4 × 10^7^ (0.6)	↓	1.0	ND	-	-	111-27-3	C7H14O2
25	25.3	1359	Ethyl 2-hexanoate	2	5.4 × 10^6^ (0.4)	3.9 × 10^6^ (0.3)	↓	1.4	6.1 × 10^6^ (0.3)	↑	1.1	7.8 × 10^6^ (0.6)	↑	1.4	1552-67-6	C8H14O2
26	25.5	1366	Isobutyl hexanoate	2	ND	ND	-	-	ND	-	-	2.2 × 10^6^ (0.2)	-	-	105-79-3	C10H20O2
27	25.8	1372	Ethyl lactate †	2	ND	1.2 × 10^8^ (11)	-	-	ND	-	-	1.0 × 10^8^ (5)	↓	1.2	97-64-3	C5H10O3
28	26.2	1381	Hexyl formate *	2	1.7 × 10^8^ (12)	9.3 × 10^7^ (8)	↓	1.8	2.3 × 10^8^ (10)	↑	1.4	1.1 × 10^8^ (8)	↓	1.6	629-33-4	C6H14O
29	26.6	1391	2,6-Dimethyl-5-heptenal	2	1.8 × 10^6^ (0.1)	1.2 × 10^6^ (0.1)	↓	1.5	ND	-	-	1.4 × 10^6^ (0.1)	↓	1.3	106-72-9	C9H16O
30	27.2	1402	Methyl octanoate	2	1.3 × 10^7^ (0.9)	1.3 × 10^7^ (1)	↑	1.0	7.5 × 10^6^ (0.3)	↓	1.7	1.9 × 10^7^ (1)	↑	1.5	111-11-5	C9H18O2
31	27.5	1411	trans-3-Hexen-1-ol **	2	1.4 × 10^6^ (0.1)	9.0 × 10^5^ (0.1)	↓	1.5	2.1 × 10^6^ (0.1)	↑	1.5	9.5 × 10^5^ (0.1)	↓	1.5	928-97-2	C6H12O
32	27.9	1419	3-Octanol	2	1.3 × 10^6^ (0.1)	1.1 × 10^6^ (0.1)	↓	1.2	1.5 × 10^6^ (0.1)	↑	1.1	1.0 × 10^6^ (0.1)	↓	1.3	589-98-0	C8H18O
33	28.5	1433	cis-2-Hexen-1-ol	2	1.3 × 10^6^ (0.1)	ND	-	-	1.7 × 10^6^ (0.1)	↑	1.3	ND	-	-	928-94-9	C6H12O
34	29.3	1453	Unknown 29.3449	4	ND	ND	-	-	2.3 × 10^9^ (100)	-	-	ND	-	-	-	C10H20O2
35	30.3	1476	Acetic acid **	2	2.3 × 10^8^ (17)	3.6 × 10^8^ (32)	↑	1.6	4.0 × 10^8^ (17)	↑	1.7	2.3 × 10^8^ (14)	↓	1.0	64-19-7	C2H4O2
36	30.6	1482	1-Heptanol	2	1.5 × 10^7^ (1.2)	ND	-	-	1.3 × 10^7^ (0.6)	↓	1.2	7.4 × 10^6^ (0.6)	↓	2.1	111-70-6	C7H16O
37	30.6	1482	Unknown 30.6066	4	3.6 × 10^6^ (0.3)	ND	-	-	ND	-	-	ND	-	-	-	C5H4O2
38	31.3	1499	trans-Linalool oxide (furanoid)	3	ND	ND	-	-	2.2 × 10^6^ (0.1)	-	-	ND	-	-	34995-77-2	C10H18O2
39	31.4	1503	Ethyl 7-octenoate	3	1.4 × 10^6^ (0.1)	9.7 × 10^5^ (0.1)	↓	1.5	ND	-	-	1.1 × 10^6^ (0.1)	↓	1.3	35194-38-8	C10H18O2
40	31.9	1513	2-Ethyl-1-hexanol	2	4.0 × 10^6^ (0.3)	2.1 × 10^6^ (0.2)	↓	1.9	4.9 × 10^6^ (0.2)	↑	1.2	2.7 × 10^6^ (0.2)	↓	1.5	104-76-7	C8H18O
41	32.5	1527	Geranyl ethyl ether 1	3	1.0 × 10^6^ (0.1)	7.3 × 10^5^ (0.1)	↓	1.4	ND	-	-	1.0 × 10^6^ (0.1)	↓	1.0	1000285-27-5	C12H22O
42	32.8	1536	1-Octanol	2	8.1 × 10^6^ (0.6)	2.9 × 10^6^ (0.3)	↓	2.8	1.1 × 10^7^ (0.5)	↑	1.3	3.2 × 10^6^ (0.3)	↓	2.5	111-87-5	C8H18O
43	33.2	1545	2-Nonanol	2	6.8 × 10^6^ (0.5)	6.2 × 10^6^ (0.6)	↓	1.1	7.8 × 10^6^ (0.3)	↑	1.1	5.7 × 10^6^ (0.4)	↓	1.2	628-99-9	C9H20O
44	33.5	1552	Ethyl nonanoate	2	8.4 × 10^6^ (0.6)	6.6 × 10^6^ (0.6)	↓	1.3	ND	-	-	ND	-	-	123-29-5	C11H22O2
45	34.2	1569	Ethyl 2-hydroxy-4-methylpentanoate	2	1.1 × 10^7^ (0.8)	7.5 × 10^6^ (0.7)	↓	1.4	7.0 × 10^6^ (0.3)	↓	1.5	5.9 × 10^6^ (0.5)	↓	1.8	10348-47-7	C8H16O3
46	34.2	1570	β-Linalool	2	4.8 × 10^6^ (0.4)	5.1 × 10^6^ (0.5)	↑	1.1	5.4 × 10^6^ (0.2)	↑	1.1	5.0 × 10^6^ (0.3)	↑	1.0	78-70-6	C10H18O
47	34.4	1575	2,3-Butanediol	2	8.1 × 10^7^ (6)	9.7 × 10^7^ (9)	↑	1.2	1.2 × 10^8^ (5)	↑	1.5	1.1 × 10^8^ (8)	↑	1.3	513-85-9	C4H10O2
48	34.8	1583	Unknown 34.7591	4	2.8 × 10^7^ (2)	1.9 × 10^7^ (2)	↓	1.5	2.4 × 10^7^ (1)	↓	1.2	1.9 × 10^7^ (1)	↓	1.5	-	C8H18O
49	34.9	1588	Unknown 34.9582	4	7.9 × 10^5^ (0.1)	2.6 × 10^5^ (0.0)	↓	3.0	7.0 × 10^5^ (0.0)	↓	1.1	ND	-	-	-	-
50	35.3	1596	Unknown 35.2873	4	2.0 × 10^7^ (2)	8.8 × 10^6^ (0.8)	↓	2.3	2.4 × 10^7^ (1)	↑	1.2	9.5 × 10^6^ (0.6)	↓	2.1	-	C8H16O3
51	35.9	1611	Unknown 35.8931	4	2.6 × 10^7^ (2)	3.5 × 10^7^ (3)	↑	1.4	3.9 × 10^7^ (2)	↑	1.5	3.5 × 10^7^ (2)	↑	1.4	-	C6H12O2
52	36.5	1627	Propylene Glycol	3	5.4 × 10^6^ (0.4)	4.4 × 10^6^ (0.4)	↓	1.2	6.5 × 10^6^ (0.3)	↑	1.2	5.5 × 10^6^ (0.4)	↑	1.0	57-55-6	C3H8O2
53	37.0	1642	Unknown 37.0675	4	2.7 × 10^6^ (0.2)	2.3 × 10^6^ (0.2)	↓	1.2	3.8 × 10^6^ (0.1)	↑	1.4	ND	-	-	-	C12H24O
54	37.7	1657	Ethyl decanoate	2	7.5 × 10^8^ (55)	ND	-	-	3.8 × 10^8^ (17)	↓	2.0	1.6 × 10^9^ (100)	↑	2.1	110-38-3	C12H24O2
55	38.1	1668	4-methyl-benzaldehyde	3	ND	ND	-	-	1.2 × 10^6^ (0.1)	-	-	ND	-	-	104-87-0	C8H8O
56	38.5	1678	Isoamyl octanoate *	2	1.7 × 10^7^ (1)	1.0 × 10^7^ (0.9)	↓	1.7	8.7 × 10^6^ (0.4)	↓	2.0	2.0 × 10^7^ (2)	↑	1.2	2035-99-6	C13H26O2
57	38.8	1686	1-nonanol *	2	1.8 × 10^7^ (1)	1.0 × 10^7^ (0.9)	↓	1.7	2.3 × 10^7^ (1)	↑	1.3	9.8 × 10^6^ (0.8)	↓	1.8	143-08-8	C9H20O
58	39.0	1691	Unknown 38.9824	4	1.5 × 10^6^ (0.1)	ND	-	-	1.9 × 10^6^ (0.8)	↑	1.3	ND	-	-	-	C15H32
59	39.2	1696	Unknown 39.1966	4	9.2 × 10^6^ (0.7)	6.1 × 10^6^ (0.5)	↓	1.5	1.0 × 10^7^ (0.4)	↑	1.1	7.0 × 10^6^ (0.5)	↓	1.3	-	C5H10O2
60	39.4	1702	Diethyl succinate	2	1.3 × 10^9^ (94)	2.9 × 10^8^ (26)	↓	4.3	ND	-	-	3.5 × 10^8^ (21)	↓	3.6	123-25-1	C8H14O4
61	39.7	1710	Ethyl 9-decenoate	3	2.0 × 10^7^ (2)	1.8 × 10^7^ (2)	↓	1.1	2.1 × 10^7^ (0.9)	↑	1.0	3.2 × 10^7^ (2)	↑	1.6	67233-91-4	C12H22O2
62	40.3	1725	α-Terpineol †	2	ND	1.7 × 10^6^ (0.2)	-	-	5.9 × 10^6^ (0.3)	↑	3.5	1.6 × 10^6^ (0.1)	↓	1.0	98-55-5	C10H18O
63	40.8	1738	Unknown 40.7705	2	4.5 × 10^6^ (0.3)	1.8 × 10^6^ (0.2)	↓	2.5	5.6 × 10^6^ (0.2)	↑	1.2	2.6 × 10^6^ (0.2)	↓	1.7	-	C12H16O2
64	41.0	1744	2-Undecanol	3	ND	ND	-	-	1.6 × 10^6^ (0.1)	-	-	ND	-	-	1653-30-1	C11H24O
65	41.1	1746	3-(methylthio)-1-Propanol	3	4.5 × 10^6^ (0.3)	2.7 × 10^6^ (0.2)	↓	1.7	5.0 × 10^6^ (0.2)	↑	1.1	3.4 × 10^6^ (0.3)	↓	1.3	505-10-2	C4H10OS
66	41.3	1754	Unknown 41.3476	4	1.4 × 10^6^ (0.1)	ND	-	-	2.1 × 10^6^ (0.1)	↑	1.5	ND	-	-	-	C11H12O3
67	41.5	1758	Unknown 41.5185 *	4	1.7 × 10^6^ (0.1)	5.0 × 10^5^ (0.0)	↓	3.5	8.8 × 10^5^ (0.0)	↓	2.0	9.0 × 10^5^ (0.1)	↓	1.9	-	C13H16
68	41.8	1767	Octyl ether †	3	ND	7.5 × 10^5^ (0.1)	-	-	ND	-	-	8.9 × 10^5^ (0.1)	↑	1.2	629-82-3	C16H34O
69	42.2	1776	trans-4-(1,1-dimethylethyl)-cyclohexanol	3	ND	1.2 × 10^6^ (0.1)	-	-	ND	-	-	ND	-	-	21862-63-5	C10H20O
70	42.6	1787	Decyl alcohol	2	9.4 × 10^6^ (0.7)	9.8 × 10^6^ (0.9)	↑	1.0	9.1 × 10^6^ (0.4)	↓	1.0	9.7 × 10^6^ (0.8)	↑	1.0	112-30-1	C10H22O
71	42.7	1790	Unknown 42.6886	2	3.8 × 10^7^ (3)	3.3 × 10^7^ (3)	↓	1.1	1.9 × 10^7^ (0.8)	↓	2.0	1.8 × 10^7^ (1)	↓	2.1	-	C8H9NO2
72	42.9	1794	Methyl Salicylate	2	ND	ND	-	-	1.3 × 10^6^ (0.1)	-	-	ND	-	-	119-36-8	C8H8O3
73	43.3	1807	Ethyl phenylacetate	2	2.3 × 10^6^ (0.2)	7.0 × 10^5^ (0.1)	↓	3.3	3.3 × 10^6^ (0.2)	↑	1.4	8.2 × 10^5^ (0.1)	↓	2.8	101-97-3	C10H12O2
74	43.7	1818	Unknown 43.7419	4	4.5 × 10^6^ (0.3)	1.8 × 10^6^ (0.2)	↓	2.5	3.7 × 10^6^ (0.2)	↓	1.2	2.1 × 10^6^ (0.1)	↓	2.1	-	C8H14O4
75	44.1	1828	Unknown 44.0789	4	1.0 × 10^6^ (0.1)	6.2 × 10^5^ (0.1)	↓	1.7	ND	-	-	7.5 × 10^5^ (0.1)	↓	1.4	-	C14H22
76	44.4	1839	Phenethyl acetate *	2	1.5 × 10^7^ (1)	1.3 × 10^7^ (1)	↓	1.2	2.7 × 10^7^ (1)	↑	1.8	1.5 × 10^7^ (0.8)	↑	1.0	103-45-7	C10H12O2
77	44.7	1846	β-Damascenone	2	3.5 × 10^6^ (0.3)	5.8 × 10^6^ (0.5)	↑	1.6	4.0 × 10^6^ (0.2)	↑	1.1	7.1 × 10^6^ (0.6)	↑	2.0	23726-93-4	C13H18O
78	45.3	1863	Ethyl dodecanoate	2	2.7 × 10^7^ (2)	2.9 × 10^7^ (3)	↑	1.1	1.7 × 10^7^ (0.8)	↓	1.5	2.4 × 10^7^ (2)	↓	1.1	106-33-2	C14H28O2
79	45.6	1872	Hexanoic acid *	2	5.6 × 10^7^ (4)	3.3 × 10^7^ (3)	↓	1.7	5.9 × 10^7^ (3)	↑	1.1	5.7 × 10^7^ (3)	↑	1.0	142-62-1	C6H12O2
80	46.0	1883	Isoamyl decanoate *	2	2.4 × 10^6^ (0.2)	1.1 × 10^6^ (0.1)	↓	2.1	1.1 × 10^6^ (0.1)	↓	2.3	1.6 × 10^6^ (0.1)	↓	1.5	2306-91-4	C15H30O2
81	46.7	1902	Benzyl alcohol ***	2	1.3 × 10^7^ (0.9)	7.3 × 10^6^ (0.6)	↓	1.8	2.2 × 10^7^ (1)	↑	1.7	6.4 × 10^6^ (0.5)	↓	2.0	100-51-6	C7H8O
82	47.0	1912	Unknown 47.0219	2	6.7 × 10^5^ (0.1)	6.0 × 10^5^ (0.1)	↓	1.1	7.0 × 10^5^ (0.0)	↑	1.1	ND	-	-	-	C16H30O4
83	47.6	1929	Ethyl 3-methylbutyl succinate	3	4.7 × 10^7^ (3)	1.8 × 10^7^ (2)	↓	2.7	4.4 × 10^7^ (2)	↓	1.1	2.8 × 10^7^ (2)	↓	1.7	28024-16-0	C11H20O4
84	48.0	1940	2-Phenylethanol	2	1.4 × 10^9^ (100)	1.1 × 10^9^ (100)	↓	1.2	ND	-	-	1.3 × 10^9^ (42)	↓	1.1	60-12-8	C8H10O
85	48.9	1967	Unknown 48.8990	4	ND	ND	-	-	6.8 × 10^6^ (0.3)	-	-	ND	-	-	-	C7H5ClF3N
86	49.7	1991	1-Dodecanol	2	2.3 × 10^6^ (0.2)	2.4 × 10^6^ (0.2)	↑	1.0	2.4 × 10^6^ (0.1)	↑	1.0	3.2 × 10^6^ (0.2)	↑	1.4	112-53-8	C12H26O
87	50.9	2032	Diphenyl ether	2	ND	ND	-	-	ND	-	-	2.5 × 10^5^ (0.0)	-	-	101-84-8	C12H10O
88	51.7	2062	4-Ethylguaiacol	3	ND	ND	-	-	1.8 × 10^6^ (0.1)	-	-	ND	-	-	2785-89-9	C9H12O2
89	52.0	2072	Unknown 51.9854	2	7.6 × 10^5^ (0.1)	1.4 × 10^6^ (0.1)	↑	1.8	8.6 × 10^5^ (0.0)	↑	1.1	1.5 × 10^6^ (0.1)	↑	1.9	-	C15H26O3
90	52.1	2078	Ethyl tetradecanoate	3	7.0 × 10^5^ (0.1)	1.2 × 10^6^ (0.1)	↑	1.7	3.8 × 10^5^ (0.0)	↓	1.8	6.4 × 10^5^ (0.0)	↓	1.1	124-06-1	C16H32O2
91	52.3	2084	Unknown 52.3099	4	ND	ND	-	-	4.6 × 10^5^ (0.0)	-	-	ND	-	-	-	C10H20O2
92	52.5	2093	Octanoic acid	2	1.2 × 10^8^ (9)	9.3 × 10^7^ (8)	↓	1.3	1.3 × 10^8^ (6)	↑	1.1	1.8 × 10^8^ (9)	↑	1.5	124-07-2	C8H16O2
93	54.4	2194	Unknown 54.4273	4	2.3 × 10^6^ (0.1)	1.7 × 10^6^ (0.1)	↓	1.4	ND	-	-	ND	-	-	-	C8H7NO2
94	54.5	5197	4-Ethylphenol	2	ND	ND	-	-	3.5 × 10^7^ (2)	-	-	ND	-	-	123-07-9	C8H10O
95	55.8	2274	Ethyl hexadecanoate	2	2.3 × 10^6^ (0.2)	1.1 × 10^6^ (0.1)	↓	2.0	1.7 × 10^6^ (0.1)	↓	1.4	1.3 × 10^6^ (0.1)	↓	1.8	628-97-7	C18H36O2
96	56.1	2288	Unknown 56.0757	4	ND	ND	-	-	ND	-	-	1.7 × 10^5^ (0.0)	-	-	-	C16H18
97	56.3	2301	Decanoic acid	2	7.7 × 10^6^ (0.6)	1.0 × 10^7^ (0.9)	↑	1.4	6.1 × 10^6^ (0.3)	↓	1.3	1.9 × 10^7^ (1)	↑	2.5	334-48-5	C10H20O2
98	56.5	2313	Unknown 56.5471	4	8.2 × 10^5^ (0.1)	5.6 × 10^5^ (0.1)	↓	1.5	7.3 × 10^5^ (0.0)	↓	1.1	7.3 × 10^5^ (0.1)	↓	1.1	-	C13H14ClF2NO3
99	56.8	2324	2,4-Di-tert-butylphenol	2	1.2 × 10^7^ (0.9)	2.4 × 10^6^ (0.2)	↓	4.8	1.3 × 10^7^ (0.6)	↑	1.1	2.7 × 10^6^ (0.2)	↓	4.3	96-76-4	C14H22O
100	57.4	2354	Ethyl trans-2-butenoate	2	ND	ND	-	-	2.0 × 10^7^ (0.9)	-	-	ND	-	-	56-81-5	C3H8O3
101	58.9	2426	Ethyl hydrogen succinate	3	8.6 × 10^6^ (0.6)	ND	-	-	ND	-	-	ND	-	-	1070-34-4	C6H10O4

RT: retention time (min); RI: retention index; Ab: abundance; RelAb: relative abundance; ND: not detected; Id: identification level (1 = identified compounds, 2 = putatively annotated compounds, 3 = putatively characterized compound classes, 4 = unknown compounds—see Sumner et al., 2007); Rel Ab: relative abundance; FC: fold change against LaChanga2017, † against LaChanga2018, (↑ = upstream, ↓ = downstream); * *p* < 0.05, ** *p* < 0.01, *** *p* < 0.001.

**Table 2:** Decisive metabolites for vintage differentiation.

RI	Name	Abundance (Area)		Fold Change		DataBase ID	Aromatic Properties ^1^
		2017	2018				
901	Ethyl acetate	1,048,176,640	640,235,900	↓	2	YMDB00569	Anise, balsam, ethereal
1084	Ethyl 3-methylbutyrate	23,746,934	5,515,695	↓	4	YMDB16003	Apple, Fruity, pineapple
1261	Styrene ^†^	3,931,121	6,247,420	↓	1	YMDB16080	Balsam, floral, plastic
1381	Hexyl formate	197,498,208	99,272,832	↓	2	HMDB0032874	Present in fruits
1402	Methyl octanoate ^†^	9,709,568	15,677,219	↓	1	YMDB01339	Aldehydic, green, herbal
1411	trans-3-Hexen-1-ol	1,722,258	927,801	↓	2	YMDB01421	Green, cortex, leafy
1419	3-Octanol	1,443,536	1,057,637	↓	1	HMDB0030070	Earthy, mushroom, dairy
1513	2-Ethyl-1-hexanol	4,465,255	2,385,446	↓	2	YMDB01330	Citrus, floral, fresh,
1536	1-Octanol	9,221,439	3,018,129	↓	3	YMDB00808	Aldehyde, burnt, chemical
1583	Unknown 34.7591	25,799,506	18,890,604	↓	1	-	-
1596	Unknown 35.2873	22,104,118	9,159,423	↓	2	-	-
1686	1-nonanol	20,248,112	10,009,123	↓	2	YMDB15917	Bitter, fatty, floral
1696	Unknown 39.1966	9,593,656	6,539,898	↓	1	-	-
1738	Unknown 40.7705	4,980,681	2,160,692	↓	2	-	-
1746	3-Methylthio-1-propanol	4,779,528	2,999,897	↓	2	YMDB1427	Widely distributed aroma constituent of foods and beverages.
1807	Ethyl phenylacetate	2,776,861	755,631	↓	4	HMDB0032618	Apricot, banana, brandy
1818	Unknown 43.7419	4,071,294	1,918,293	↓	2	-	-
1846	β-Damascenone ^†^	3,772,885	6,413,788	↓	1	YMDB15908	Apple, honey, rose
1902	Benzyl alcohol	16,751,528	6,809,303	↓	2	YMDB01426	Balsamic, cherry, floral
1929	Ethyl 3-methylbutyl succinate	45,479,080	22,022,998	↓	2	CID119794	Found in wine and beer
2072	Unknown 51.9854 ^†^	810,844	1,414,013	↑	2	-	-
2274	Ethyl hexadecanoate	1,946,713	1,206,513	↓	2	YMDB01349	Balsam, creamy, fruity
2301	Decanoic acid ^†^	6,862,970	14,093,453	↑	2	YMDB00677	Citrus, fatty, rancid
2324	2,4-Di-tert-butylphenol	12,312,931	2,577,305	↓	5	YMDB15942	Phenolic

RI: retention index; ↓: downstream, ↑: upstream; YMDB: Yeast Metabolome Database; HMDB: Human Metabolome Database; CID: PubChem Compound ID number. ^1^ Sources: Found in indicated database and/or in The Good Scents Company Information System (at: http://www.thegoodscentscompany.com, accessed on 6 November 2019); ^†^ PC1-negative loadings: related to 2018 vintage; not indicated are PC1-positive loadings: related to 2017 vintage.

**Table 3:** Data-acquisition parameters.

Extraction HS-SPME Fiber 50/30 μm DVB/CAR/PDMS		Detector MS-QToF	
Sample	Conditioning at 40 °C/5 min Extraction at 40 °C/30 min	Ion source	Electron ionization (EI)
		Source Temperature	230 °C
Fiber conditioning	Pre-extraction at 250 °C/10 min Post-desorption at 250 °C/5 min	Emission energy	15.2 μA
		Electron energy	70 eV
Desorption	240 °C/10 min	Data storage	Profile
Separation GC column DB-WAX 30 m/250 μm/0.25 μm		Solvent delay	3 min
		Quadropole TT1 cutoff mass	30 amu
Inlet mode	Splitless		
Flow rate	1.0 mL/min He (RT Locked 2-Undecanone at 36.158 min)	Mass range	30 to 400 amu
		Acquisition rate	2.5 spectra/s
Oven	40 °C for 5 min	Acquisition time	400 ms/spectrum
	3 °C/min to 180 °C	Transients/spectrum	5443
	30 °C/min to 220 °C for 10 min	System with backflush and gas saver mode on.	
	Total run 63 min		
Post-run	2 min at 220 °C		

## Data Availability

Metabolomics raw data are available at MetaboLights (https://www.ebi.ac.uk/metabolights/ (accessed on 6 November 2019)) with accession number MTBLS1391. CURRENTLY IN CURATION. Exact-mass library VinoST2.mslibrary.xml can be downloaded from https://www.ciad.mx/VinosMxDB (accessed on 6 November 2019).

## Supplementary Materials

The following supporting information can be downloaded online: Supplementary Material—Validated results, identified compounds in pool QC tables, batch sequence and Unknowns entities *m*/*z*; Table S1 contains repeatability, reproducibility, linearity (r), limit of detection (LOD) and limit of quantification (LOQ) determinations; Table S2 shows RT, RI, CAS# and %RSD of 74 compounds identified in pool QC; Table S3 shows the batch sequence used to acquire vineyard and vintage data; Table S4 contains *m*/*z* of 25 unknown entities detected.

## References

[1] Lacalle-Bergeron L., Izquierdo-Sandoval D., Sancho J.V., López F.J., Hernández F., Portolés T. (2021). Chromatography hyphenated to high resolution mass spectrometry in untargeted metabolomics for investigation of food (bio)markers. TrAC Trends Anal. Chem..

[2] Alañón M.E., Pérez-Coello M.S., Marina M.L. (2015). Wine science in the metabolomics era. TrAC Trends Anal. Chem..

[3] Klåvus A., Kokla M., Noerman S., Koistinen V.M., Tuomainen M., Zarei I., Meuronen T., Häkkinen M.R., Rummukainen S., Babu A.F. (2020). “Notame”: Workflow for non-targeted LC-MS metabolic profiling. Metabolites.

[4] Dunn W.B., Ellis D.I. (2005). Metabolomics: Current analytical platforms and methodologies. TrAC Trends Anal. Chem..

[5] Broadhurst D., Goodacre R., Reinke S.N., Kuligowski J., Wilson I.D., Lewis M.R., Dunn W.B. (2018). Guidelines and considerations for the use of system suitability and quality control samples in mass spectrometry assays applied in untargeted clinical metabolomic studies. Metabolomics.

[6] Spicer R.A., Salek R., Steinbeck C. (2017). Comment: A decade after the metabolomics standards initiative it’s time for a revision. Sci. Data.

[7] Sumner L.W., Amberg A., Barrett D., Beale M.H., Beger R., Daykin C.A., Fan T.W.-M., Fiehn O., Goodacre R., Griffin J.L. (2007). Proposed minimum reporting standards for chemical analysis. Metabolomics.

[8] Baran R. (2017). Untargeted metabolomics suffers from incomplete raw data processing. Metabolomics.

[9] Beger R.D., Dunn W.B., Bandukwala A., Bethan B., Broadhurst D., Clish C.B., Dasari S., Derr L., Evans A., Fischer S. (2019). Towards quality assurance and quality control in untargeted metabolomics studies. Metabolomics.

[10] Dudzik D., Barbas-bernardos C., García A., Barbas C. (2018). Quality assurance procedures for mass spectrometry untargeted metabolomics a review. J. Pharm. Biomed. Anal..

[11] Dunn W.B., Broadhurst D.I., Edison A., Guillou C., Viant M.R., Bearden D.W., Beger R.D. (2017). Quality assurance and quality control processes: Summary of a metabolomics community questionnaire. Metabolomics.

[12] Martin J.C., Maillot M., Mazerolles G., Verdu A., Lyan B., Migné C., Defoort C., Canlet C., Junot C., Guillou C. (2015). Can we trust untargeted metabolomics? Results of the metabo-ring initiative, a large-scale, multi-instrument inter-laboratory study. Metabolomics.

[13] Capece A., Romaniello R., Siesto G., Pietrafesa R., Massari C., Poeta C., Romano P. (2010). Selection of indigenous saccharomyces cerevisiae strains for nero d’avola wine and evaluation of selected starter implantation in pilot fermentation. Int. J. Food Microbiol..

[14] Robinson A.L., Boss P.K., Heymann H., Solomon P.S., Trengove R.D. (2011). Development of a sensitive non-targeted method for characterizing the wine volatile profile using headspace solid-phase microextraction comprehensive two-dimensional gas chromatography time-of-flight mass spectrometry. J. Chromatogr. A.

[15] Arapitsas P., Scholz M., Vrhovsek U., Di Blasi S., Biondi A., Masuero D., Perenzoni D., Rigo A., Mattivi F. (2012). A metabolomic approach to the study of wine micro-oxygenation. PLoS ONE.

[16] Arapitsas P., Speri G., Angeli A., Perenzoni D., Mattivi F. (2014). The influence of storage on the ‘chemical age’ of red wines. Metabolomics.

[17] López-Rituerto E., Savorani F., Avenoza A., Busto J.H., Peregrina J.M., Engelsen S.B. (2012). Investigations of la rioja terroir for wine production using 1H NMR metabolomics. J. Agric. Food Chem..

[18] Castro C.C., Martins R.C., Teixeira J.A., Ferreira A.C.S. (2014). Application of a high-throughput process analytical technology metabolomics pipeline to port wine forced ageing process. Food Chem..

[19] Silva Ferreira A.C., Monforte A.R., Silva Teixeira C., Martins R., Fairbairn S., Bauer F.F. (2014). Monitoring alcoholic fermentation: An untargeted approach. J. Agric. Food Chem..

[20] Alves Z., Melo A., Figueiredo A.R., Coimbra M.A., Gomes C., Rocha S.M. (2015). Exploring the saccharomyces cerevisiae volatile metabolome: Indigenous versus commercial strains. PLoS ONE.

[21] Boss P.K., Kalua C.M., Nicholson E.L., Maffei S.M., Böttcher C., Davies C. (2018). Fermentation of grapes throughout development identifies stages critical to the development of wine volatile composition. Aust. J. Grape Wine Res..

[22] Brown M., Dunn W.B., Ellis D.I., Goodacre R., Handl J., Knowles J.D., O’Hagan S., Spasić I., Kell D.B. (2005). A metabolome pipeline: From concept to data to knowledge. Metabolomics.

[23] Jones C.M., Dunn W.B., Raftery D., Hartung T., Wilson I.D., Lewis M.R., Tayyari F., Baljit K., Souza A., Ntai I. (2018). Metabolomics Quality Assurance and Quality Control Consortium (MQACC): Reference and Test Material Working Group.

[24] Bletsou A.A., Jeon J., Hollender J., Archontaki E., Thomaidis N.S. (2015). Targeted and non-targeted liquid chromatography-mass spectrometric workflows for identification of transformation products of emerging pollutants in the aquatic environment. TrAC Trends Anal. Chem..

[25] Martínez-Bueno M.J., Gómez Ramos M.J., Bauer A., Fernández-Alba A.R. (2019). An Overview of non-targeted screening strategies based on high resolution accurate mass spectrometry for the identification of migrants coming from plastic food packaging materials. TrAC Trends Anal. Chem..

[26] Palermo A., Botre F., de la Torre X., Zamboni N. (2017). Non-targeted LC-MS based metabolomics analysis of the urinary steroidal profile. Anal. Chim. Acta.

[27] Muñoz-Redondo J.M., Puertas B., Pereira-Caro G., Ordóñez-Díaz J.L., Ruiz-Moreno M.J., Cantos-Villar E., Moreno-Rojas J.M. (2021). A statistical workflow to evaluate the modulation of wine metabolome and its contribution to the sensory attributes. Fermentation.

[28] Covarrubias J., Thach L. (2015). Wines of Baja Mexico: A qualitative study examining viticulture, enology, and marketing practices. Wine Econ. Policy.

[29] Larios Córdova H. (2016). Iniciativa Con Proyecto de Decreto Que Expide La Ley General de Fomento a La Industria Vitivinícola. https://infosen.senado.gob.mx/sgsp/gaceta/63/2/2017-04-25-1/assets/documentos/Inic_PAN_Ley_Industria_Vitivinicila.pdf.

[30] OIV Estadísticas de México de 1995 a 2016. https://www.oiv.int/es/statistiques/recherche.

[31] DOF El Pleno del Senado Aprobó la Ley General de Fomento a la Industria Vitivinícola. http://comunicacion.senado.gob.mx/index.php/informacion/boletines/39130-el-pleno-del-senado-aprobo-la-ley-general-de-fomento-a-la-industria-vitivinicola.html.

[32] CMV Marca Colectiva. https://vinomexicano.org.mx/marca-colectiva/.

[33] FDA Bioanalytical Method Validation, Guidance for Industry. https://www.fda.gov/media/70858/download.

[34] Dashko S., Zhou N., Tinta T., Sivilotti P. (2015). Use of non-conventional yeast improves the wine aroma profile of Ribolla Gialla. J. Ind. Microbiol. Biotechnol..

[35] Parker M., Capone D.L., Francis I.L., Herderich M.J. (2017). Aroma precursors in grapes and wine: Flavor release during wine production and consumption. J. Agric. Food Chem..

[36] Ramirez-Gaona M., Marcu A., Pon A., Guo A.C., Sajed T., Wishart N.A., Karu N., Feunang Y.D., Arndt D., Wishart D.S. (2017). YMDB 2.0: A significantly expanded version of the yeast metabolome database. Nucleic Acids Res..

[37] Martins N., Garcia R., Mendes D., Costa Freitas A.M., da Silva M.G., Cabrita M.J. (2018). An ancient winemaking technology: Exploring the volatile composition of amphora wines. LWT Food Sci. Technol..

[38] Lu Y., Sun F., Wang W., Liu Y., Wang J., Sun J., Mu J., Gao Z. (2020). Effects of spontaneous fermentation on the microorganisms diversity and volatile compounds during ‘Marselan’ from grape to wine. LWT Food Sci. Technol..

[39] Lu Y., Guan X., Li R., Wang J., Liu Y., Ma Y., Lv J., Wang S., Mu J. (2021). Comparative study of microbial communities and volatile profiles during the inoculated and spontaneous fermentation of persimmon wine. Process Biochem..

[40] Varsha K.K., Devendra L., Shilpa G., Priya S., Pandey A., Nampoothiri K.M. (2015). 2,4-Di-Tert-Butyl phenol as the antifungal, antioxidant bioactive purified from a newly isolated *Lactococcus* sp.. Int. J. Food Microbiol..

[41] Yin L., Wang C., Zhu X., Ning C., Gao L., Zhang J., Wang Y., Huang R. (2020). A multi-step screening approach of suitable non-saccharomyces yeast for the fermentation of hawthorn wine. LWT Food Sci. Technol..

[42] Mendes B., Gonalves J., Câmara J.S. (2012). Effectiveness of high-throughput miniaturized sorbent- and solid phase microextraction techniques combined with gas chromatography-mass spectrometry analysis for a rapid screening of volatile and semi-volatile composition of wines—A comparative study. Talanta.

[43] Câmara J.S., Arminda Alves M., Marques J.C. (2006). Development of headspace solid-phase microextraction-gas chromatography-mass spectrometry methodology for analysis of terpenoids in Madeira wines. Anal. Chim. Acta.

[44] Hjelmeland A.K., King E.S., Ebeler S.E., Heymann H. (2013). Characterizing the chemical and sensory profiles of United States cabernet sauvignon wines and blends. Am. J. Enol. Vitic..

[45] Wylie P., Hjelmeland A., Runnebaum R., Ebeler S. (2016). Analysis of pinot noir wines by HS-SPME GC/Q-TOF: Correlating geographical origin with volatile aroma profiles. Planta Med..

[46] Baumann S., Conjelko T., Aronova S., Lafond S., David F., Ebeler S.E. Accurate mass retention time locked flavor database by GC-TOF. Proceedings of the American Society for Mass Spectrometry Annual Conference.

