# Supplementary material for: Characterization of Cabernet Sauvignon Wines by Untargeted HS-SPME GC-QTOF-MS
Source: Molecules. 2022 Mar 7;27(5):1726. doi: 10.3390/molecules27051726 (PMC8911954; doi:10.3390/molecules27051726)
Supplement: Supplementary file 1 [file molecules-27-01726-s001.zip › molecules-1573464-supplementary.pdf]

Characterization of Cabernet Sauvignon wines by untargeted HS-SPME GC-QTOF/MS

Alejandra Chávez-Márquez[1], Alfonso A. Gardea[1], Humberto González-Rios[1] and Luz Vazquez-Moreno[1,*]

[1] Centro de Investigación en Alimentación y Desarrollo A.C. Carretera Gustavo Enrique Astiazarán Rosas, No. 46, Col. La Victoria, CP. 83304, Hermosillo, Sonora, México; alejandra.chavez@estudiantes.ciad.mx (ACM); gardea@ciad.mx (AAG); hgory@ciad.mx (HGR)

* Correspondence: lvazquez@ciad.mx

Supplementary Table S1. Repeatability, Reproducibility, Linearity (*r*), Limit of detection (LOD) and limit of quantification (LOQ) determinations.
Supplementary Table S2. Identified compounds in pool QC.
Supplementary Table S3. Mass to charge ratio of unknown components.
Supplementary Table S4. Batch sequence.

Supplementary Table S1. Repeatability, Reproducibility, Linearity (*r*), Limit of detection (LOD) and limit of quantification (LOQ) determinations.

Standards	Level 1	Level 2	Level 3	Level 4	Level 5	PW	LOD	LOQ	*r*
α-Pinene (ng/L)	1.5	3.0	6.2	12.5	25.0	ND	0.16	0.55	0.9998
Area (mean ± SD)[1]	$1.7x10^7±7.3x10^6$	$3.3x10^7±1.7x10^6$	$7.3x10^7±3.4x10^6$	$1.4x10^8±9x10^6$	$2.8x10^8±1.93x10^7$				$y=1x10^7x-3x10^6$
Repeatability (%RSD)[1]	4	5	5	7	1				
Reproducibility (%RSD)[2]	13	-	5	-	7				
RT (10.66 ± 0.01)[3]									
β-Pinene (ng/L)	1.5	3.0	6.2	12.5	25.0	ND	1.70	2.35	0.9992
Area (mean ± SD)[1]	$1.2x10^7±2x10^6$	$2.5x10^7±4.5x10^6$	$5.3x10^7±5.4x10^6$	$1x10^8±1.2x10^7$	$2.1x10^8±9.4x10^6$				$y=1x10^7x-3x10^6$
Repeatability (%RSD)[1]	16	18	10	11	4				
Reproducibility (%RSD)[2]	15	-	12	-	18				
RT (13.91 ± 0.01)[3]									
p-Cymene (ng/L)	0.3	0.6	1.5	2.5	5.0	ND	0.10	0.33	0.9965
Area (mean ± SD)[1]	$1.2x10^7±2x10^6$	$2.3x10^7±2.3x10^6$	$4.6x10^7±3.9x10^6$	$1x10^8±1.9x10^7$	$1.8x10^8±1.4x10^7$				$y=3x10^7x+4x10^6$
Repeatability (%RSD)[1]	13	10	8	19	7				
Reproducibility (%RSD)[2]	11	-	8	-	12				
RT (21.22 ± 0.09)[3]									
2-Undecanone (ng/L)	0.3	0.6	1.5	2.5	5.0	ND	0.04	0.15	0.9998
Area (mean ± SD)[1]	$3.8x10^7±7.2x10^6$	$6.7x10^7±6.2x10^6$	$1.3x10^7±1.3x10^6$	$2.9x10^8±3.5x10^7$	$6x10^8±5.2x10^7$				$y=1x10^7x-1x10^5$
Repeatability (%RSD)[1]	10	9	9	12	8				
Reproducibility (%RSD)[2]	15	-	9	-	10				
RT (36.19 ± 0.02)[3]									

LOD and LOQ n=10; [1]n=3; [2]n=6; [3]RT=Retention time in minutes, n=24. PW= pooled wines as blank sample.

Supplementary Table S2. Identified compounds in pool QC.

Component RT	Component RI	Delta RI	CAS#	Compound Name	Match Factor	Component Area	Mean	SD	%RSD[1]	Rel. Ab.[2]	Library[3]
6.74	899.72	1.14	141-78-6	Ethyl Acetate	71.79	1087566213.59	1100750853.81	66285968.86	6.02	100.0000	1
7.20	915.50	2.19	67-56-1	Methyl Alcohol	98.10	13587391.37	15013483.34	469947.94	3.13	1.3639	1
9.07	979.62	ND	97-62-1	Ethyl isobutyrate	85.96	15584492.68	17165442.35	1257693.09	7.33	1.5594	2
11.52	1050.51	0.39	105-54-4	Ethyl butyrate	99.63	44572541.31	47038254.97	1107987.17	2.36	4.2733	1
11.99	1062.40	1.18	71-23-8	1-Propanol	89.58	16519309.31	20014759.34	1616279.19	8.08	1.8183	1
12.19	1067.23	0.28	7452-79-1	Ethyl α-methylbutyrate	98.12	12364596.67	14518819.58	334954.94	2.31	1.3190	1
12.84	1083.51	0.85	108-64-5	Ethyl isovaleriate	99.59	25416351.31	26706195.80	315372.66	1.18	2.4262	1
13.21	1092.92	0.39	-	Unknown 13.2265	77.91	606649.29	944230.04	102967.10	10.90	0.0858	1
14.61	1126.43	0.57	78-83-1	Isobutyl alcohol	99.76	87994861.16	104833869.14	1698484.22	1.62	9.5239	1
15.00	1134.94	0.26	123-92-2	Isoamyl acetate	99.18	204784005.40	217565827.57	4227683.76	1.94	19.7652	1
20.91	1263.61	-2.91	100-42-5	Styrene	94.75	2185898.78	2637408.69	230436.37	8.74	0.2396	1
21.36	1273.26	-0.03	106-27-4	Isoamyl butyrate	84.82	4501969.30	4311124.89	401591.32	9.32	0.3917	1
21.88	1284.42	ND	142-92-7	Hexyl acetate	74.51	4215107.49	6644252.36	696714.22	10.49	0.6036	2
22.70	1302.11	-0.71	1000450-02-5	Furfuryl ethyl ether	90.48	1454541.77	1780045.02	80808.85	4.54	0.1617	1
23.24	1313.96	ND	513-86-0	Acetoin	87.05	5123806.82	6406296.59	343083.10	5.36	0.5820	3
24.08	1332.80	0.52	-	Unknown 24.1025	75.06	805323.58	684910.56	103863.08	15.16	0.0622	1
24.53	1342.98	0.23	626-89-1	4-Methyl-1-pentanol	78.16	4007739.36	4799090.32	254606.05	5.31	0.4360	1
24.65	1345.78	ND	106-30-9	Ethyl heptanoate	84.34	8158810.48	10845075.98	1235292.79	11.39	0.9852	2
25.05	1354.65	1.02	111-27-3	1-Hexanol	99.28	11354719.15	12568021.68	182580.94	1.45	1.1418	1
25.31	1360.62	-1.62	1552-67-6	Ethyl 2-hexanoate	91.96	5488715.71	6345702.45	313644.20	4.94	0.5765	1
25.82	1372.03	0.25	97-64-3	Ethyl lactate	95.64	152996004.42	189613393.54	11923776.76	6.29	17.2258	1
26.19	1380.53	0.64	629-33-4	Hexyl formate	99.88	148708934.43	166283152.83	3400573.41	2.05	15.1063	1
26.60	1389.73	1.12	106-72-9	2,6-Dimethyl-5-heptenal	84.34	2739757.24	2199600.34	181051.22	8.23	0.1998	1
27.11	1401.17	0.95	111-11-5	Methyl octanoate	89.96	7524103.30	9065134.37	522206.22	5.76	0.8235	1
27.50	1409.98	1.29	928-97-2	trans-3-Hexen-1-ol	75.79	1547391.82	1526070.25	71385.97	4.68	0.1386	1
27.90	1419.17	-0.08	589-98-0	3-Octanol	90.26	988144.89	1389772.62	70707.22	5.09	0.1263	1

28.47	1432.61	0.28	928-94-9	cis-2-Hexen-1-ol	88.09	1060782.93	1272632.77	46475.68	3.65	0.1156	1
30.29	1475.04	0.80	64-19-7	Acetic acid	99.08	207322164.46	276816056.07	31130368.46	11.25	25.1479	1
30.61	1482.42	-0.10	-	Unknown 30.6066	96.09	1978989.65	2989409.36	99746.27	3.34	0.2716	1
31.51	1503.48	ND	35194-38-8	Ethyl 7-octenoate	86.86	945796.35	1161878.55	101158.12	8.71	0.1056	3
31.94	1513.49	-0.40	104-76-7	2-Ethyl-1-hexanol	95.93	5231903.24	6700126.80	496535.62	7.41	0.6087	1
32.84	1535.81	-0.28	111-87-5	1-Octanol	95.93	12316994.51	15903671.30	273784.79	1.72	1.4448	1
33.19	1544.36	0.59	628-99-9	2-Nonanol	97.22	5467682.51	7104505.80	631315.64	8.89	0.6454	1
33.53	1552.89	-0.92	123-29-5	Ethyl nonanoate	98.36	9350689.12	11068694.60	911506.44	8.23	1.0056	1
34.22	1569.84	-0.67	10348-47-7	Ethyl 2-hydroxy-4-mthylpentanoate	77.43	6464520.07	9982126.20	390297.61	3.91	0.9068	1
34.24	1570.49	-0.05	78-70-6	ß-Linalool	98.07	3961162.19	5309272.77	510920.38	9.62	0.4823	1
34.40	1574.50	0.85	513-85-9	2,3-Butanediol	96.32	77184019.72	93491060.57	6755710.08	7.23	8.4934	1
34.75	1582.98	0.31	-	Unknown 34.7591	98.79	20412970.14	26028322.52	624778.38	2.40	2.3646	1
34.94	1587.79	0.43	-	Unknown 34.9582	90.25	506305.29	729575.37	28302.78	3.88	0.0663	1
35.29	1596.47	-0.11	-	Unknown 35.2873	98.06	13207988.42	18319788.04	499196.19	2.72	1.6643	1
35.43	1599.92	0.63	-	Unknown 35.4563	91.05	1047785.33	1919098.78	126593.28	6.60	0.1743	1
35.88	1610.99	0.38	-	Unknown 35.8931	98.79	23698502.24	28229852.54	1932323.25	6.84	2.5646	1
36.48	1626.45	ND	57-55-6	Propylene Glycol	91.25	4299517.72	5109297.02	341953.17	6.69	0.4642	3
37.70	1657.88	-0.59	110-38-3	Ethyl decanoate	99.28	578242990.82	692663626.49	56769634.66	8.20	62.9265	1
38.54	1679.49	-1.30	2035-99-6	Isoamyl octanoate	97.14	10410589.81	14809788.69	1204182.62	8.13	1.3454	1
38.80	1686.24	-0.27	143-08-8	1-nonanol	99.59	17179953.44	25494378.22	737472.70	2.89	2.3161	1
38.99	1691.08	-0.18	-	Unknown 38.9824	77.93	1255935.02	2080581.02	149834.50	7.20	0.1890	1
39.20	1696.44	-0.03	-	Unknown 39.1966	91.01	7157775.48	9966946.86	550827.24	5.53	0.9055	1
39.74	1710.52	ND	67233-91-4	Ethyl 9-decenoate	94.64	15241111.88	18500131.85	1378250.24	7.45	1.6807	3
40.71	1736.45	1.64	-	Unknown 40.7705	92.97	3527214.72	5733993.98	309131.38	5.39	0.5209	1
41.07	1746.06	ND	505-10-2	γ-Methylmercaptopropyl alcohol	91.49	3306390.14	3586758.48	363625.05	10.14	0.3258	3
41.30	1752.44	1.16	-	Unknown 41.3476	94.41	1492670.86	2408209.86	151986.66	6.31	0.2188	1
41.47	1756.79	1.40	-	Unknown 41.5185	97.62	1170441.35	1403476.86	69958.39	4.98	0.1275	1
42.60	1787.37	-0.11	112-30-1	Decyl alcohol	87.32	7609909.11	10934867.02	604886.57	5.53	0.9934	1

42.68	1789.55	0.10	-	Unknown 42.6886	97.85	9210521.72	20910866.93	4250821.59	20.33	1.8997	1
42.88	1794.71	-0.65	119-36-8	Methyl Salicylate	88.49	669786.21	932393.10	30375.33	3.26	0.0847	1
43.35	1807.35	-0.51	101-97-3	Ethyl phenylacetate	97.40	2241946.39	3194437.22	145990.75	4.57	0.2902	1
43.76	1818.83	-0.40	-	Unknown 44.0789	70.29	559929.51	5166464.97	312417.85	6.05	0.4694	1
44.48	1839.35	-0.67	103-45-7	Phenethyl acetate	99.24	13727297.93	19136937.94	599374.11	3.13	1.7385	1
44.72	1846.18	-0.62	23726-93-4	ß-Damascenone	95.22	2509843.92	3736854.57	183153.42	4.90	0.3395	1
45.32	1863.47	-0.62	106-33-2	Ethyl dodecanoate	99.59	19477196.39	33662540.00	2396767.09	7.12	3.0581	1
45.62	1871.93	0.18	142-62-1	Hexanoic acid	98.37	29416030.48	47472921.33	1542840.11	3.25	4.3128	1
46.00	1882.71	0.20	2306-91-4	Isoamyl decanoate	94.33	2468695.08	2192428.21	186893.66	8.52	0.1992	1
46.67	1901.78	0.41	100-51-6	Benzyl alcohol	99.62	17715301.28	18232514.84	966158.77	5.30	1.6564	1
47.02	1911.79	0.08	-	Unknown 47.0219	80.05	692648.87	892203.28	76701.60	8.60	0.0811	1
47.64	1930.09	ND	28024-16-0	Ethyl isopentyl succinate	98.10	34103106.14	51661101.74	2106330.70	4.08	4.6933	3
48.92	1967.55	-0.49	-	Unknown 48.8990	98.07	2155690.47	2782961.23	102370.40	3.68	0.2528	1
49.70	1990.60	0.50	112-53-8	1-Dodecanol	98.87	1998429.52	2166370.42	314933.04	14.54	0.1968	1
51.75	2063.28	ND	2785-89-9	4-Ethylguaiacol	85.28	233959.13	377778.62	23751.78	6.29	0.0343	3
51.99	2072.48	-0.35	-	Unknown 51.9854	86.76	602160.62	910808.57	80437.26	8.83	0.0827	1
52.17	2079.16	ND	124-06-1	Ethyl tetradecanoate	75.65	229063.00	783095.85	84269.98	10.76	0.0711	3
52.53	2092.74	0.42	124-07-2	Octanoic acid	99.30	134073301.73	111872820.53	14154425.41	12.65	10.1633	1
53.23	2124.61	ND	128-37-0	Butylated Hydroxytoluene	70.71	553769.07	775200.05	103260.37	13.32	0.0704	3
54.50	2197.98	-0.57	123-07-9	4-Ethylphenol	96.38	6228561.18	9184470.82	252967.57	2.75	0.8344	1
55.83	2273.47	0.06	628-97-7	Ethyl hexadecanoate	95.13	2216880.16	2154547.35	307475.32	14.27	0.1957	1
56.29	2299.69	1.08	334-48-5	Decanoic acid	96.91	9970396.21	6334453.35	880603.38	13.90	0.5755	1
56.55	2313.62	-0.35	-	Unknown 56.5471	89.91	684971.89	953279.07	54680.84	5.74	0.0866	1
56.76	2323.67	-0.11	96-76-4	2,4-Di-tert-butylphenol	97.02	2400361.89	2526980.47	219728.25	8.70	0.2296	1
58.89	2426.47	ND	1070-34-4	Monoethyl succinate	81.85	6568825.59	9519758.90	1324171.21	13.91	0.8648	3

[1]Reproducibility, n=15; [2]Relative abundance of identified peaks; [3]1= VinoST2.mslibrary.xml, 2=Flavors-14.mslibrary.xml, 3=NIST 17; ND=Not determined

Supplementary Table S3. Mass to charge ratio of unknown components

#	RT	RI	Compound	m/z	Formula*
8	13.228	1093	Unknown 13.2265	103.0749, 71.0853, 75.0440, 69.0698, 47.0129	$C_9H_{20}O_2$
12	16.578	1170	Unknown 16.5912	105.0699, 121.1009, 93.0700, 136.1246, 91.0543	$C_{10}H_{16}$
21	23.918	1328	Unknown 23.8754	71.0490, 99.0802, 69.0697, 41.0387, 93.0696	$C_{10}H_{18}O$
34	29.288	1453	Unknown 29.3449	101.05960, 70.0414, 57.0701, 127.1114, 60.0208	$C_{10}H_{20}O_2$
37	30.610	1482	Unknown 30.6066	96.02010, 95.0125, 67.0178, 96.0382, 96.0527	$C_5H_4O_2$
48	34.762	1583	Unknown 34.7591	56.06220, 69.0699, 55.0545, 70.0776, 41.0389	$C_8H_{18}O$
49	34.943	1588	Unknown 34.9582	74.01820, 148.0543, 61.0105, 151.0234, 75.0255	-
50	35.289	1596	Unknown 35.2873	45.03370, 43.0544, 70.0777, 71.0851, 55.0543	$C_8H_{16}O_3$
51	35.898	1611	Unknown 35.8931	45.03380, 57.0337, 43.0181, 47.0494, 45.0978	$C_6H_{12}O_2$
53	37.045	1642	Unknown 37.0675	57.03360, 43.0543, 41.0387, 55.0543, 67.0541	$C_{12}H_{24}O$
58	38.994	1691	Unknown 38.9824	43.05430, 71.0852, 57.0699, 70.0774, 55.0543	$C_{15}H_{32}$
59	39.200	1696	Unknown 39.1966	60.02070, 74.0362, 41.0388, 57.0699, 87.0438	$C_5H_{10}O_2$
63	40.765	1738	Unknown 40.7705	192.15010, 177.1266, 149.0957, 131.085, 105.0695	$C_{12}H_{16}O_2$
66	41.339	1754	Unknown 41.3476	163.11170, 145.1009, 164.1163, 73.0647, 121.1011	$C_{11}H_{12}O_3$
67	41.533	1758	Unknown 41.5185	157.10080, 142.077, 172.1242, 141.0695, 158.1041	$C_{13}H_{16}$
71	42.691	1790	Unknown 42.6886	133.01350, 151.024, 134.0139, 134.9926, 152.0244	$C_8H_9NO_2$
74	43.744	1818	Unknown 43.7419	101.02320, 129.0543, 57.0699, 73.0283, 56.0619	$C_8H_{14}O_4$
75	44.084	1828	Unknown 44.0789	190.1340, 107.0848, 105.0692, 175.1107, 91.0538	$C_{14}H_{22}$
82	47.025	1912	Unknown 47.0219	71.04890, 43.0542, 83.0853, 56.0619, 69.0697	$C_{16}H_{30}O_4$
85	48.905	1967	Unknown 48.8990	174.99940, 147.9884, 195.0056, 176.9963, 149.9854	$C_7H_5ClF_3N$
89	51.992	2072	Unknown 51.9854	85.0280, 69.0696, 41.0386, 93.0691, 71.0488	$C_{15}H_{26}O_3$
91	52.316	2084	Unknown 52.3099	71.08530, 87.0437, 88.0515, 102.0671, 57.0697	$C_{10}H_{20}O_2$
93	54.425	2194	Unknown 54.4273	149.04410, 105.0692, 104.0615, 133.0128, 150.0449	$C_8H_7NO_2$
96	56.080	2288	Unknown 56.0757	181.09980, 165.0682, 166.0757, 210.1386, 167.0832	$C_{16}H_{18}$
98	56.550	2313	Unknown 56.5471	91.0541, 176.0832, 103.0541, 121.0646, 92.0613	$C_{13}H_{14}ClF_2NO_3$

RT: Retention Time; RI: Retention Index; m/z: mass to charge ratio; *Proposed formula

Supplementary Table S4. Batch sequence

Line	Type	Rack/Vials	DataFile	Sample Name
1)	MassCal:	'MSTOFMASSCAL(PAUSEFAIL)'		
2)	Sample	6/1	BcoST_03	BcoST
3)	Sample	6/2	FortST_03	FortST
4)	Sample	6/3	LaChanga_2017_02	LaChanga_2017
5)	MassCal:	'MSTOFMASSCAL(PAUSEFAIL)'		
6)	Sample	6/4	LosDolores_2018_01	LosDolores_2018
7)	Sample	6/5	BcoST_02	BcoST
8)	Sample	6/6	LaChanga_2018_03	LaChanga_2018
9)	MassCal:	'MSTOFMASSCAL(PAUSEFAIL)'		
10)	Sample	6/7	LosDolores_2018_02	LosDolores_2018
11)	Sample	6/8	FortST_01	FortST
12)	Sample	6/9	LaChanga_2017_03	LaChanga_2017
13)	MassCal:	'MSTOFMASSCAL(PAUSEFAIL)'		
14)	Sample	6/10	LaChanga_2017_01	LaChanga_2017
15)	Sample	6/11	LosDolores_2017_02	LosDolores_2017
16)	Sample	6/12	LosDolores_2017_03	LosDolores_2017
17)	MassCal:	'MSTOFMASSCAL(PAUSEFAIL)'		
18)	Sample	6/13	LosDolores_2017_01	LosDolores_2017
19)	Sample	6/14	LosDolores_2018_03	LosDolores_2018
20)	Sample	6/15	LaChanga_2018_01	LaChanga_2018
21)	MassCal:	'MSTOFMASSCAL(PAUSEFAIL)'		
22)	Sample	9/1	FortST_02	FortST
23)	Sample	9/2	LaChanga_2018_02	LaChanga_2018
24)	Sample	9/3	BcoST_01	BcoST

BcoST= Pool, FortST= Pool spiked, MassCal= keyword to perform mass calibration and stop sequence if it failed.
